# An international collaborative approach to learning histology using a virtual microscope

**DOI:** 10.1111/ahe.12888

**Published:** 2022-11-14

**Authors:** Sue‐Mian Then, Marie Kokolski, Yvonne Mbaki, Deborah Merrick, Susan Anderson

**Affiliations:** ^1^ Division of Biomedical Sciences University of Nottingham Malaysia; ^2^ School of Medicine University of Nottingham UK; ^3^ School of Life Sciences University of Nottingham UK

**Keywords:** histology, microanatomy, undergraduate education, virtual microscope

## Abstract

Histology is often taught in higher education settings using online virtual microscopes (VM). This study aimed to develop and evaluate the use of VM in teaching on a BSc degree at the University of Nottingham by surveying students and staff. A key development was the use of an e‐workbook so that students were actively engaged in creating their own bespoke revision material. Subsequently, this approach was used in a second study evaluating the use of VM in teaching the histology and pathology of the gastrointestinal (GI) tract via group work with students from two BSc courses at the University of Nottingham; one based at Derby (RDHC) and the other in Malaysia (UNMC). Students worked together in groups to complete an e‐workbook, develop a presentation, and decide how to collaborate and communicate. An evaluation of these activities revealed advantages in developing transferrable skills, and good engagement with both the histology topic and group work. Analysis of assessment of the module at UNMC showed that student performance improved in the histology‐based module after the intervention (*p* < 0.01) and that this improvement was not evident in other modules taken by the cohort. Furthermore, when interrogating the questions from the examination paper that asked students to identify features from histological images, fewer questions were seen as ‘difficult’ (*p* < 0.001) and more were seen as ‘average’ (*p* < 0.01). This study demonstrates that the use of VM in histology combined with active learning in creating a revision resource enhances engagement and depth of learning. When further combined with collaborative active group work, students developed a range of histology knowledge and transferrable skills, with notable improvement in examination performance relative to other contemporaneous modules.

## 1 INTRODUCTION

The subject of histology is fundamentally important when it comes to informing understanding of normal tissue structure and the pathological basis of disease. Since the middle of the 19th century, histology was traditionally taught in microscopy laboratories (Dee, 2009). Specifically, students accessed sets of slides with individual microscopes and teachers projected slides onto screens, which allowed for commentary and orientation of the material. Typically, additional staff or postgraduate student demonstrators provided supplementary support for these often‐large sessions.

Over the last decade with the introduction of virtual microscopy (VM), histology teaching has been revolutionised, opening a myriad of educational opportunities including more flexible and inclusive learning environments. VM became possible with the creation of virtual slides in the mid‐1980s (Dee, 2009)by systematically scanning entire conventional microscope slides at high magnification (typically with a ×40 objective) and creating a digital montage by stitching individual fields of view together. The virtual slide was viewed at a variety of magnifications, and as an overview or thumbnail image of the entire slide (Dee, 2009). The image can be zoomed and panned to mimic the experience of using a microscope, with the use of tiling software similar to Google Earth, which ensured the rapid loading of high‐resolution data often through an on‐line platform. The advantages of this new digital histology era have been well documented, including the flexibility to access the material and ease of magnification and orientation within the specimen (Collier et al., 2012; Gatumu et al., 2014; Saco et al., 2016). Students were often more comfortable with this mode of learning, empowering them to talk through what they can see more readily with the teacher and access material independently outside of the practical setting (Anyanwu et al., 2012; Braun & Kearns, 2008; Wilson et al., 2016). That said, there is the perception from some academics that students who do not learn how to use a conventional light microscope lack important practical skills, including the appreciation of specimen scale (Scoville & Buskirk, 2007), though most have embraced the advantages. Specific limitations including the maintenance of microscopes and adequate slide collection, as well as the space it takes to house such collections is not negligible. Therefore, utilising VM has increasingly become more mainstream in a range of educational settings and globally, with many universities making the complete transition from optical to virtual microscopy (Fernanda et al., 2021; Fernandes et al., 2018; Krippendorf & Lough, 2005; Vainer et al., 2017; Wu & Chiang, 2022). With pedagogical meta‐analysis revealing that the VM methodology is slightly superior to conventional optical microscopy, the switch in teaching practices has become an obvious choice for many (Chang et al., 2021; Lee, 2020; Mione et al., 2013; Wilson et al., 2016). The use of VM alongside complementary online technologies became essential to educators and students during the pandemic, where lockdown and social distancing measures prevented the running of in‐person histology sessions(Caruso, 2021).Some universities reported having capacity to run fully digital histology courses during the pandemic with the use of VM and video‐conferencing platforms, whilst others adopted the VM to teach students confocal and electron microscopy techniques (Lionetti, 2022).Such novel developments in the learning environment highlight the importance of VM as part of the technical advances observed in histology education in the 21^st^ century (Darici et al., 2021; Sharma et al., 2021).

For the purposes of this article, VM refers to the use of interactive digitised slides, for histology and histopathology teaching. This article aims to(a) Explore the development and subsequent evaluation of utilising a virtual microscope for histology teaching, with considerations of the benefits and limitations of VM from both the teacher and student perspective. Within this, the purpose is to offer a description of the developments surrounding the use of VM as part of maximising engagement, active learning, and revision by the students, (b) Develop and evaluate an innovative international group histology project.

## 2 METHODS

### 2.1 Study 1: Developing and evaluating a virtual microscope for histology teaching

Virtual microscopy (VM) was introduced in the Division of Medical Sciences and Graduate Entry Medicine at the University of Nottingham School of Medicine, Royal Derby Hospital Centre (RDHC) to provide histology teaching to students enrolled on a new BSc programme in Medical Physiology and Therapeutics (MPT). Because this was a new programme of study, students were introduced to VM from the beginning, with no prior use of microscopes and slides in the course. VM sessions were incorporated into all modules so that histology was taught in an integrated way as students learnt about each body system. Each VM session was preceded with a didactic one‐hour lecture covering the histological structure of the relevant tissue/organ/system. Students then attended a VM session in groups of 25–30 with the lecturer and a demonstrator in attendance.

Students accessed the web‐based VM via the Virtual Learning environment (VLE). They also accessed a worksheet detailing the slides and tasks to be used in the session. The lecture slides were also available via VLE so that they could be referred to in the VM sessions. Each 90 min session began with a brief introduction to the tasks outlined in the worksheets and the lecturer and demonstrator spent time interacting with students throughout the session. At regular intervals throughout the lecturer conducted a summing up session.

In the first year of the degree programme there were 8 sessions (cells and tissues, alimentary, blood, blood vessels, bone and cartilage, muscle, skin, respiratory system, kidney). In the second year of the programme there were 2 sessions (nervous system and reproductive systems). Student knowledge and understanding of histology was assessed using images generated from the VM. Screenshots were generated and used as the basis for either multiple choice or extended matching examination questions, administered via the University of Nottingham e‐assessment management system, ExamSys.

Student opinion of VM sessions was evaluated by means of a questionnaire containing qualitative and quantitative questions delivered to first‐ and second‐year students. The advantages and limitations of using VM from the staff perspective were collated following an informal meeting towards the end of the academic year.

### 2.2 Study 2: Developing and evaluating an innovative international group histology project

Following on from the outcomes of study 1, study 2 took place in the following years. An e‐workbook was developed, where all students were expected to find, insert and annotate images from the VM to make their own tailored revision resources for all VM sessions across all modules. It became clear that there was potential for the use of VM, e‐workbooks and student collaboration to enhance the understanding of histology and this was pursued in collaboration with the University of Nottingham Malaysia Campus (UNMC) where there was a similar sized cohort of students studying a BSc in Biomedical Sciences (BMS). Study 2 describes the implementation and evaluation of this collaboration.

First year students studying for a BSc in MPT at the RDHC and a BSc in BMS at the UNMC took part in the international histology project from 2014–15 academic session until 2017. This collaborative teaching activity formed part of the ‘Human Development and Tissue Differentiation (HDT)’ (UNMC) and ‘Supply and Demand 1 (SD1)’ (RDHC) modules. Both cohorts of students attended a joint introductory histology lecture either physically (RDHC) or virtually (UNMC) via videoconferencing. Following the lecture, the e‐workbook was given to all students, and they were assigned to groups containing equivalent numbers of students from RDHC and UNMC and assigned a specific region of the GI tract to focus on, for example oesophagus, stomach, small intestine, large intestine, anal canal. Students were asked to contact their group members, introduce themselves and carry out an ice‐breaking activity. The communication platform the students used to contact each other was not prescribed, but students generally opted for University email or Facebook groups. How tasks were completed, and by whom in the group, was left for the group to consider and implement. In this way, the module was designed to develop groupwork skills of time management, group dynamics, and communication. Contact details for the course convenors (UNMC and RDHC) were given to all students if they required further support or had difficulties working in the allocated group. All groups were advised that collaborative working to ensure all members of the group succeed was the best way to emulate a real workplace environment and conclude a successful project.

Students worked as international groups to collectively complete an e‐workbook on a region of the GI tract. Students were able to access the virtual microscope at any point during the project ensuring all students irrespective of their physical locations were able to access the same material. There were three tasks included in the workbook. Firstly, groups identified the different layers seen in cross‐sections at various points throughout the GI tract and described the function. Secondly, groups identified the various types and function of the epithelium from the different parts of the GI tract and considered the impact of this on tissue function. Finally, groups chose from a selection of common diseases affecting the region of the GI tract and explained how the alteration in structure led to altered function and, therefore, symptoms of the pathology.

Students used this information to develop a 10 min group presentation; to include the normal histology, the alterations in microstructure of a chosen pathology of the area and the consequent impact of this disrupted structure on function. They could also comment on how the disease might then manifest symptoms in a patient, or be diagnosed via a biopsy, for example. Presentations were delivered by students to their peers within their own campus, so that all groups had the opportunity to hear presentations on all GI regions. Feedback was provided by academic colleagues and peer interactions via questions was encouraged. Peer assessment was also incorporated into the presentation session, so that students provided and received anonymous feedback from each other on their presentation. The workbook was not part of the summative assessment.

**FIGURE 1:**
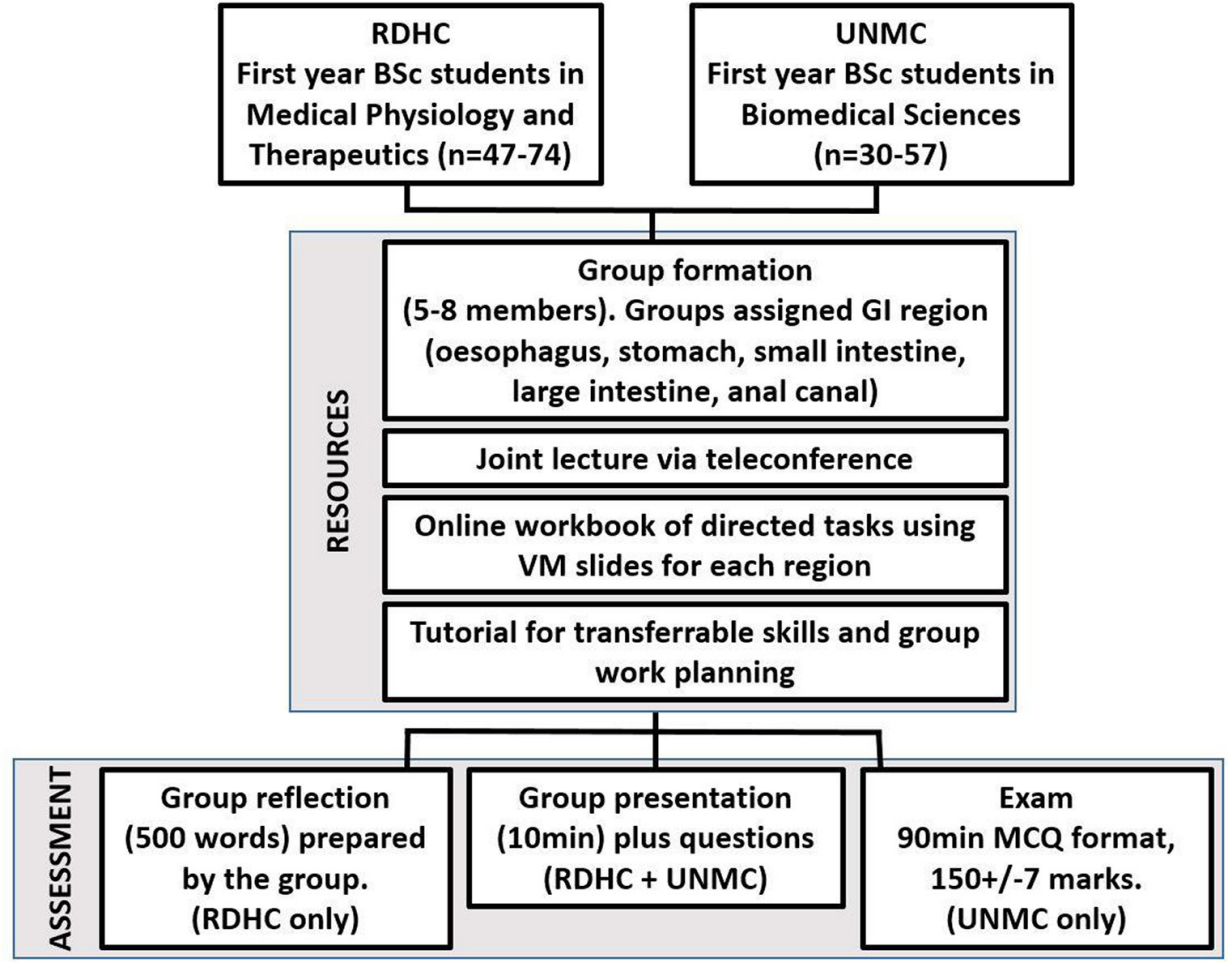
Outline of the International histology project and assessment.

The international histology (IH) project was assessed as demonstrated in Figure 1.

Quantitative analysis was performed by analysing the Year 1 UNMC student cohort performance in the A11HDT (human development and tissue differentiation) module before and after the IH project. Furthermore, a comparison was made between student performance on the A11HDT module and the other Year 1 modules (listed in Table 1). Further analysis of student performance in questions utilising identification of histology features was done using the difficulty index and discrimination analysis provided within ExamSys.

**TABLE 1:** Modules taken by Year 1 Biomedical Sciences students from 2014–2017 at UNMC with summative ExamSys assessment

Module Code	Module Name
A11HDT	Human Development and Tissue Differentiation
A11MBM	Molecular Basis of Medicine
B11M01	Physiology and Pharmacology 1
B11M02	Physiology and Pharmacology 2
B11105	Introduction to Neuroscience

Students at RDHC had histology assessed across all first‐year modules, as it was integrated throughout, so such quantitative analyses were not possible. RDHC students completed a group reflection to consider the skills they had learnt and how they might improve in the future. Unfortunately, ethical approval was not sought for this part of the study, so is not included here. However, informal module feedback was collected and represented as a word‐cloud.

#### 2.2.1 Statistical analysis

Data were analysed and presented using GraphPad Prism. All data was normally distributed where sample sizes were large enough to test, resulting in the use of parametric analysis. Data compared as two groups were analysed using unpaired *t*‐tests. Data comparing two variables in multiple groups was analysed using two‐way ANOVA with Sidak's multiple comparisons test.

## 3 RESULTS

### 3.1 Study 1

**FIGURE 2:**
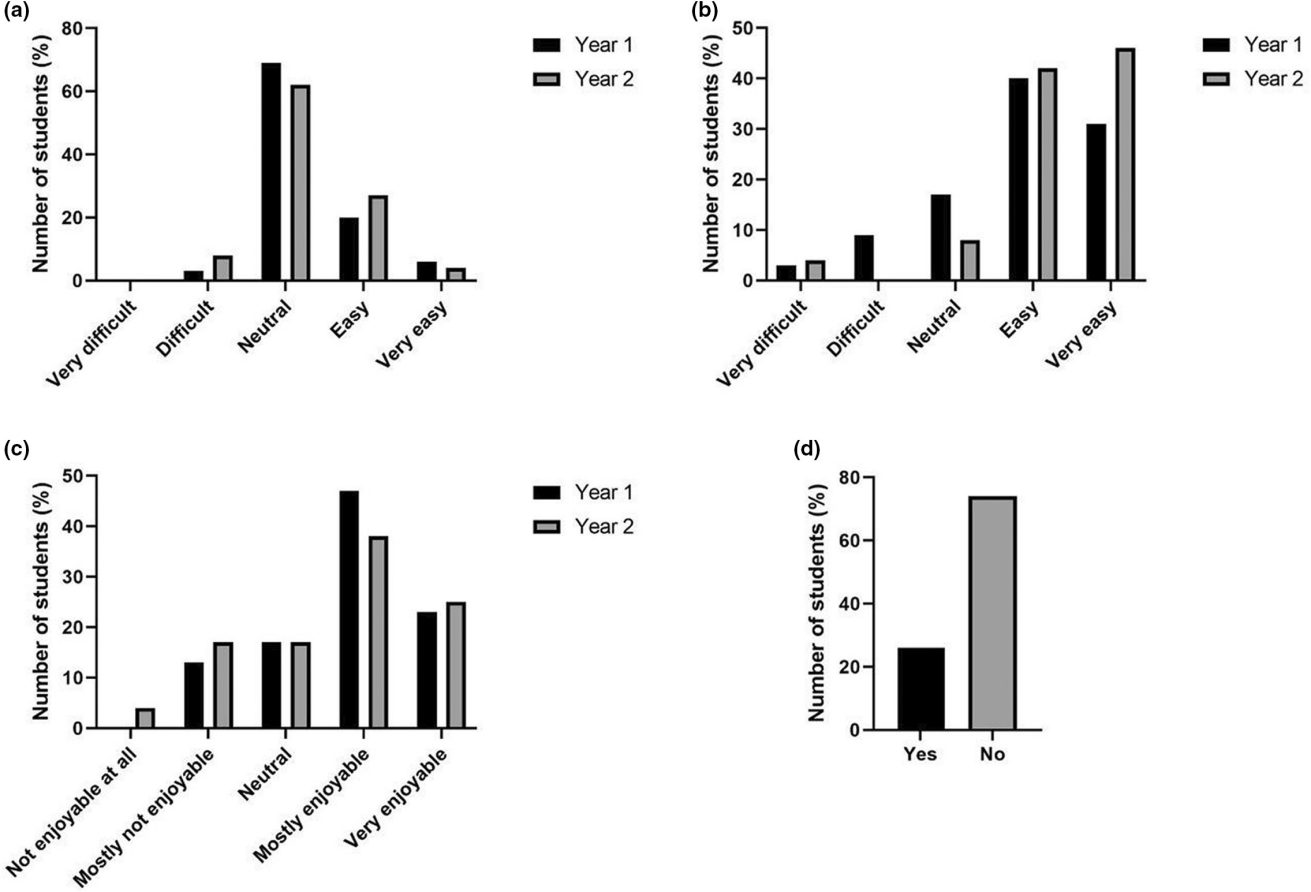
BSc MPT student opinions regarding the topic of histology and use of the virtual microscope. All data expressed as percentage of the total for that year group. Likert scale questions used for a‐c and text box question used for d. (a) How difficult do you find the subject matter of histology? year 1 *n* = 34, year 2 *n* = 26. (b) How easy is it to use the virtual microscope? year 1 *n* = 35, year 2 *n* = 26. (c) How enjoyable are the virtual microscope sessions? year 1 *n* = 30, year 2 *n* = 24. (d) Could the sessions be run entirely online (without staff)? only year 1 students were asked *n* = 31.

Survey results were generated from first‐ (*n* = 38; 75% of cohort) and second‐year year (*n* = 25; 65% of cohort) students on the BSc MPT. The quantitative analysis of BSc student evaluation of VM sessions is shown in Figure 2. Most students in both year groups found the VM ‘easy’ or ‘very easy’ to use (Figure 2b) and rated their enjoyment of the VM sessions highly (Figure 2c). This was despite the fact that students do not find the subject matter of histology easy (Figure 2a). An interesting question arose from our appraisal of the literature. If we accept that VM is comparable or superior to traditional microscopy classes and if sessions can be run entirely online, with annotations on the virtual slides, then how essential are staff‐led sessions? We asked students their opinion on whether it would be possible to run the sessions entirely as self‐directed sessions. The majority of students said that sessions could not be run adequately without staff present (Figure 2d) and those that agreed that sessions could be stand‐alone usually qualified this by saying they would need online help or recorded summing up sessions.

**FIGURE 3:**
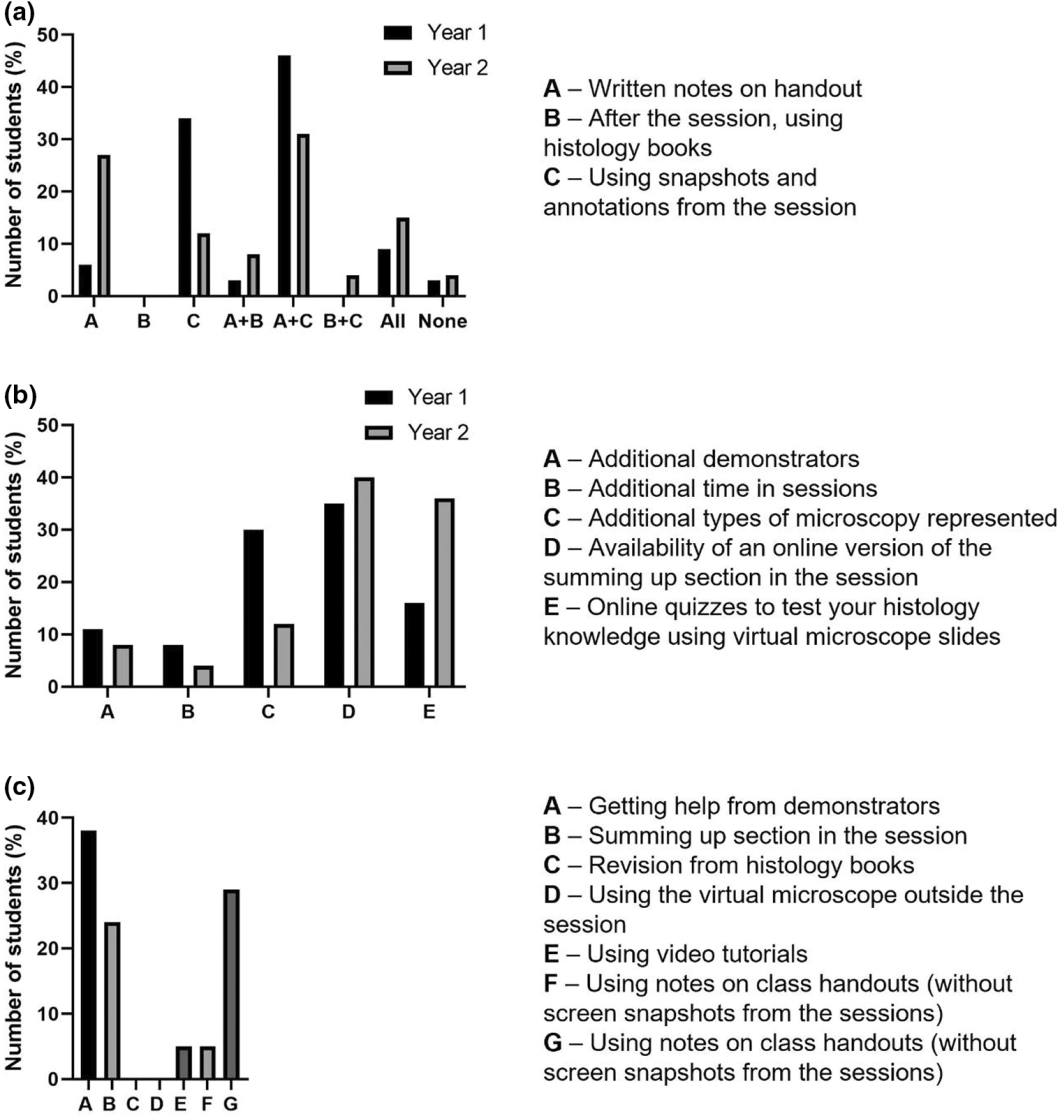
BSc MPT student opinions regarding resources related to the virtual microscope sessions. (a) How do you make notes during the virtual microscope sessions? Students had 3 options (a‐c on the figure) and could select more than one option, year 1 *n* = 35, year 2 *n* = 26. (b) Which further initiative do you think would be best to implement in the sessions? Students were given 5 options (a‐e on the figure), year 1 *n* = 37, year 2 *n* = 25. (c) What was the best beneficial resource for your examination preparation? Students were given 7 options (a‐g on the figure), only year 2 students were asked *n* = 21.

Students were asked to comment on how they made revision notes for histology (Figure 3a). Interestingly, many students chose to save screenshots from the VM and insert them into the worksheets. Students annotated and labelled their own examples for these student‐generated ‘e‐workbooks’. First year students chose this over the traditional method of drawing and labelling sketches of slides on the handouts. Students supplemented these with additional information during the summing up sessions. Furthermore, student generated e‐workbooks' were the most valuable revision tool in terms of examination performance (Figure 3c). When asked what additional resources they would like to see in the histology sessions, most students felt the session length and number of demonstrators was right and focused instead on recommending recordings of the summing up session, more types of microscopy and interactive quizzes (Figure 3b).

Student feedback on the module mentioned VM frequently and focussed on topics such as ease of use **‘Quicker and easier than ‘real’**, relatability due to being able to pan and zoom **‘better than textbook images', ‘better than seeing ‘perfect’ examples in books',** the variety of slides on offer rather than one static textbook image. The ability to keep images **‘ease of taking images away’,** and staff summing up sessions helped relate structure to function ‘**understand tissue function better’.**

**FIGURE 4:**
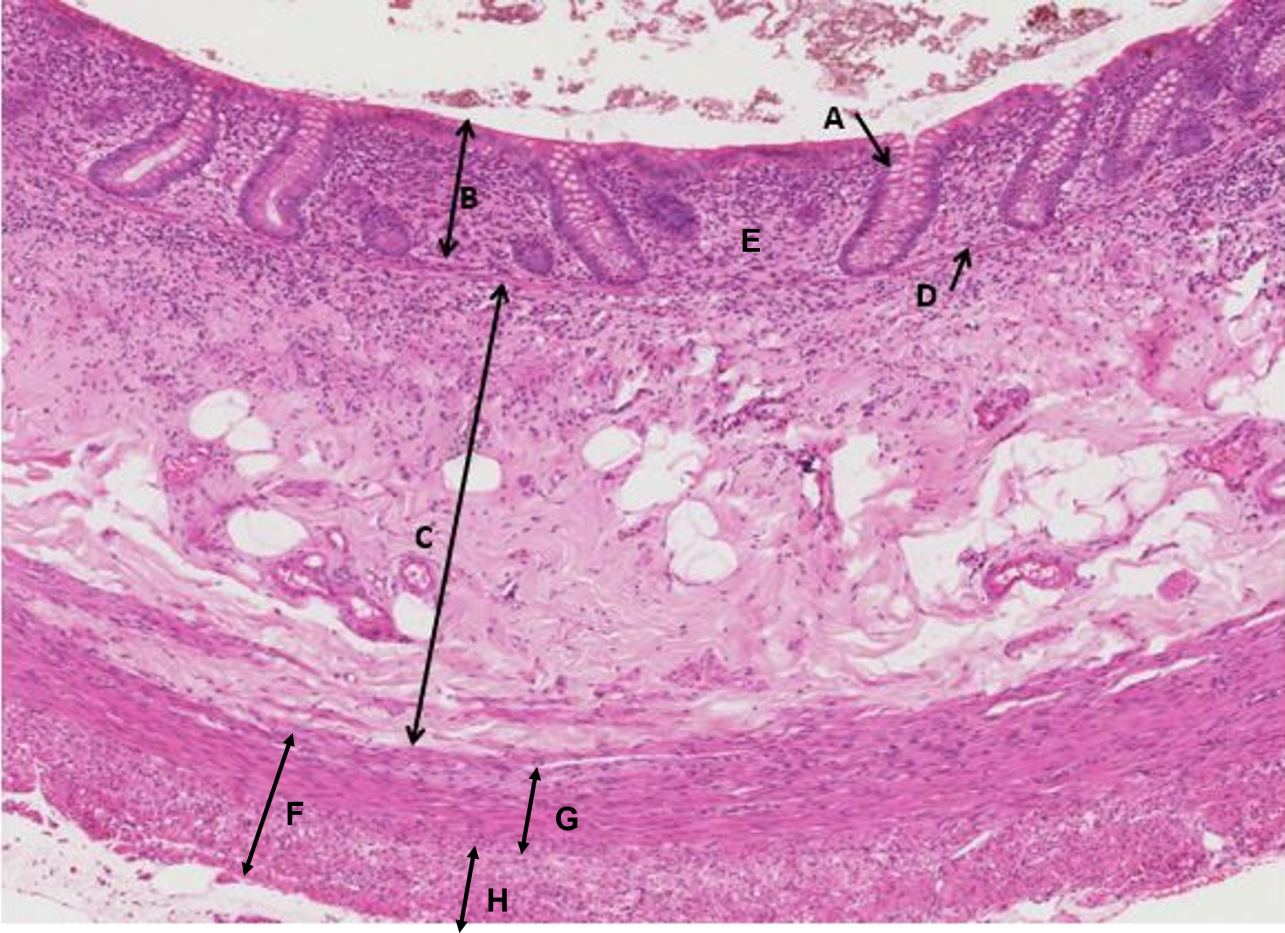
An example of an annotated screenshot from the colon that was captured from the VM and annotated and included within a student e‐workbooks. A = epithelium (goblet cell in gland); B = mucosa; C = submucosa; D = muscularis mucosa; E = lamina propria; F = muscularis propria; G = inner circular muscle layer; H = outer longditudinal muscle layer.

An informal staff feedback session allowed staff to reflect on their experiences. The following points were raised:

- The whole class could see the same slide and review as a group during summing up sessions with the class leader.
- Staff could easily see what students were looking at on screen to confirm they were looking at the right area (and not at online distractions such as Facebook)
- Staff could check understanding in an interactive way by asking students to point out and discuss features, relating structure to function
- Students worked naturally in informal groups to discuss slides. More able students helped struggling students or collaborated to get more out of the sessions
- The use of e‐workbooks engaged students actively in the sessions and students took and annotated screen shots as an alternative to making drawings (see Figure 4 for an example)
- Students had thumbnail images and scale bars to relate features to their size and location
- Students accessed lecture notes from the VLE to help identify cells and complete tasks.
- Other online resources (e.g. supplementary electron micrographs) were useful and valued.
- Access outside of timetabled sessions was higher than expected.
- The VM provided almost limitless material for the generation of examination questions

### 3.2 Study 2

**FIGURE 5:**
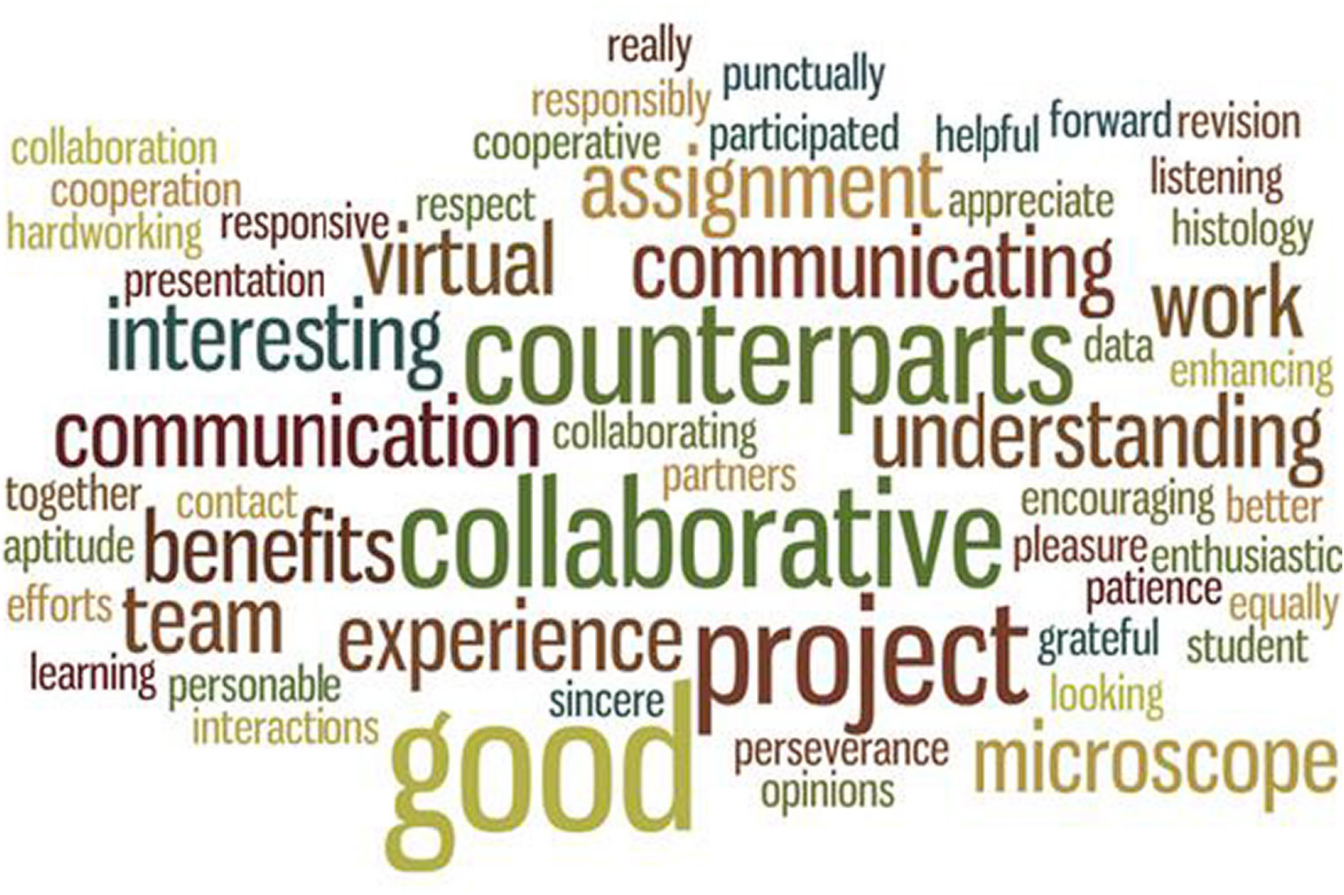
Word cloud generated from the student feedback regarding the IH project, showing the more frequent words in larger fonts. The feedback was very positive with much focus on collaboration, communication and understanding.

Analysis of the students' feedback from both campuses is shown in Figure 5 as a word cloud to illustrate the common themes from the feedback. Themes that stand out are collaboration, communication, understanding and a general appreciation of the experience. The feedback was mostly positive with the students acknowledging the value of their experience and recognising the impact of learning to negotiate and work with partners in a different time zone to form part of their collaborative learning. That said, working within a different time zone was also reported to be a limitation by the students in terms of effective engagement from both parties, in addition to competing priorities sometimes making one geographical half of a group feel that they were shouldering much of the burden.

In addition to general feedback, more specific feedback was elicited from students at UNMC. They reflected that the project helped them to understand histology better and helped them revise. All commented that the activity gave them an opportunity to apply what they had learned in the lectures and practical and they gained confidence in identifying different types of tissue under the microscope. They also commented that they had become more observant while looking under the microscope.

**FIGURE 6:**
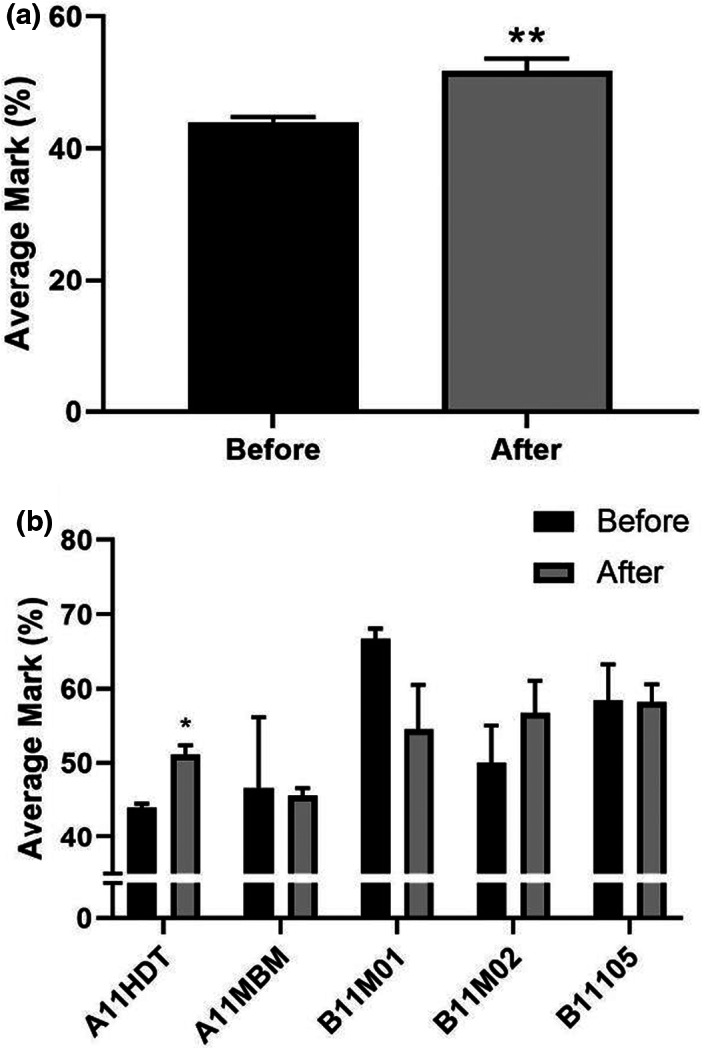
Average examination performance of students before and after the introduction of the histology teaching intervention in the A11HDT module. All data represented as mean +/− SD. (a) Average examination performance in the A11HDT module before (*n* = 2, 2012/13 and 2013/14) and after intervention (*n* = 5, 2014/15–2018/19). Statistical comparison using unpaired *t*‐test. (b) Comparison of average examination performance in all first‐year modules (module details listed in Table 1)during the time period before (*n* = 2, 2012/13 and 2013/14) and after the intervention (*n* = 3, 2014/15–2016/17). Statistical comparison using multiple unpaired *t*‐tests. * *p* < 0.05, ** *p* < 0.01.

The quantitative analysis of examination performance is based on the outcome of the UNMC students undertaking the summative assessment for the A11HDT module upon completion of the cross‐campus collaborative project. Figure 6a shows a significant increase in cumulative mean marks for the A11HDT module after the introduction of the IH project. Prior to the introduction of the IH project in 2014, the A11HDT module had one of the lowest mean scores of all Year 1 modules, indicating that students potentially encountered difficulties in translating and applying what they learnt in the histology lecture and practical sessions. Following the introduction of the IH project the A11HDT module mean score improved relative to other modules on the course (Figure 6b).

**FIGURE 7:**
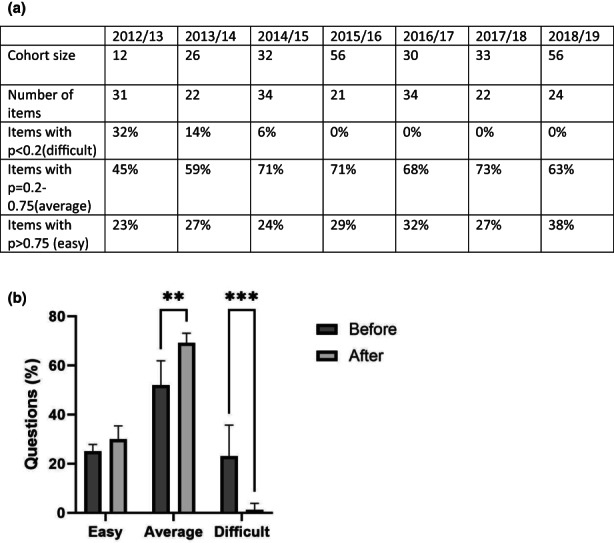
(a) Average performance of students in histology image‐based examination question in the A11HDT module from 2012–2019. An item is a question or part question worth one mark that was identified as examining knowledge of histological images. Items were identified as difficult (*p* < 0.2), average (*p* = 0.2–0.75) or easy (*p* > 0.75) based on the frequency‐discrimination analysis produced by the assessment software and are expressed as a percentage of the total number of identified items. (b) Comparison of question difficulty in the A11HDT module during the time period before (*n* = 2, 2012/13 and 2013/14) and after the intervention (*n* = 5, 2014/15–2018/19). All data represented as mean +/− SD. Statistical comparison using two‐way ANOVA and Sidak's multiple comparisons test, ** *p* < 0.01, *** *p* < 0.001

Analysis of student performance was carried out on the questions containing histology images, based on the difficulty index and discrimination analysis of the students' answer in the ExamSys examination (Figure 7a, b). This analysis indicated that students found more questions ‘easy’, significantly more questions ‘average’(*p* < 0.01) and significantly fewer questions ‘difficult’(*p* < 0.001) after the IH project was introduced.

## 4 DISCUSSION

Although VM has been available since the 1980s, limiting factors initially precluded its widespread use, for example the prohibitive cost of slide scanners and the processing speed of standard computers. Now there are at least 30 commercial vendors of scanners/software and large classes can be run in distinct locations without discernible loss of processing speed. Indeed, virtual microscopy offers numerous advantages over conventional microscopy, which have been well documented (Dee, 2009). The present study involved students enrolled on a new degree, where a comparison was not possible between the use of traditional microscopy and VM, as has been the case in other studies (Hande et al., 2017; Treanor et al., 2009). Instead, a fresh approach to learning histology was adopted. This approach demonstrated that students were able to appreciate the full range of learning structure and function of microscopic tissues without the need to master the skills of using a microscope.

The increasing use of VM has taken place alongside a concomitant reduction in the use of traditional microscopy classes over the last 2 decades. Dee (2009) reported a massive reduction in the use of microscopy laboratories and glass slides from 85% to 30% over the decade from 1997–2007. A survey of the teaching of microscopic anatomy in 45 US medical schools by Drake et al in 2009 indicated that an average of 79 h of histology teaching occurred throughout a typical course and that this was split 50–50 between lectures and practicals. This number of hours had not significantly changed since the previous survey in 2002 but the use of VM had increased from 14% to 44%.

It has been recognised that students retrieve information better if they have an active part in its generation. One way in which collaborative active learning has been facilitated is through incorporating interactive manuals or workbooks that students are guided through utilising the VM (Bloodgood, 2012; Darici et al., 2021; Felszeghy et al., 2017; Khalil et al., 2013; Sahota et al., 2016).In fact, due to the pandemic, many medical and dental schools across the world have had to adapt to using VM and video conferencing platforms with breakout room functions to foster students' interaction to proceed with the delivery of histology/histopathology courses during the lockdown periods (Darici et al., 2021; Guiter et al., 2021).In study 1 we saw that students enjoyed histology classes even though they found them difficult and the VM was easy to use. Of critical importance, student participation in generating their own learning resources increased active learning, engagement, and motivation in addition to providing a valuable revision aid. In fact, on one occasion, when the teaching staff were delayed due to a timetabling error, instructors arrived 10 min into the session to find all students engaged in the task, having begun generating their e‐workbook using the lecture slides as a guide. This indeed aligns to the literature where it was observed that the integration of VM into histology teaching increased student collaboration (Braun & Kearns, 2008; Telang et al., 2016; Triola & Holloway, 2011; Uraiby et al., 2021). The social, academic and psychological benefits of collaborative learning are widely stated in the literature and include higher productivity, engagement and achievement (Hurst et al., 2013; Laal & Ghodsi, 2012). The ability of students to see each other's screens led to a natural desire to collaborate and to cluster together in small groups to discuss what they had found. The levels of engagement were high (see comment above) and anecdotal evidence from visiting Faculty as well as staff feedback and ultimately examination results demonstrated that students' knowledge and confidence increased dramatically throughout the year. The collaborative approach also appeared to benefit weaker students who seemed comfortable to say they were struggling and seek help from their peers as well as the lecturers.

Histology is considered to be a challenging topic for students, which makes it a challenging topic for the lecturers(García et al., 2019; Sherman & Jue, 2009). A recent survey amongst undergraduate students identify several difficulties in learning histology, including lack of familiarity of terminology, complexity of the subject and lack of sufficient time (García et al., 2019). A study of first year undergraduate students found that most science and agricultural students approach their study using a surface approach, which is inclusive of memorization and is dependent on declarative knowledge to pass their examination (Santhanam et al., 1998) although they have been taught how to use concept maps in their learning. An explanation for this is the fact that the knowledge learnt by students is not sufficiently reused during the retention interval period of memory (Balemans et al., 2016).There is evidence to suggest that although students favour the easy of accessibility of the VM, they still need an interactive platform to guide them to learn as a group, as well as facilitate peer learning (García et al., 2019; Sander & Golas, 2013).This was a notable observation from the student feedback obtained within the present study.

In study 2, students benefited from the active engagement and interaction with their peers throughout the process of completing the workbook and presentation, with improvements to their grades following the implementation of this interactive IH project. It is worth acknowledging that this innovative teaching and learning modality is gaining traction in teaching histology and pathology as there is reported evidence of improvement to students' learning outcome and students' interest in the subject (Goldberg & Dintzis, 2007; Sahota et al., 2016). This environment is suitable for students to learn the complexities within histology, whilst functioning as an environment that aids in reducing the cognitive load of students through the distribution and sharing of learning activities, which can later be consolidated as information relating to a bigger picture (Lehtinen, 2003; Sahota et al., 2016). Assigning students to groups in their first semester at University may also have helped their transition through meeting new people, whilst also aiding in their development of social skills at a time when they may potentially find it difficult to integrate. Moreover, there was the benefit of disrupting potential silos through the integration of students from all backgrounds and cultures from the outset in semester one of Year 1, thus a consideration for the agenda of inclusive and equality of learning. Generally, it was perceived that students worked together effectively and inclusively as a group and they understood that encouraging all participants to reach their potential was the best way to secure a good mark in the module.

Student feedback on the cross‐campus project were largely positive as they had the opportunity to work in a truly global setting. Students recognised that they had acquired other skills such as time and group management, communication, and presentation skills. Their feedback when displayed as a word cloud focused on transferrable skills such as communication, collaboration, and other professional attributes such as patience, perseverance, listening, punctuality and responsibility as well as positive understanding and experience. Having to accommodate working in different time zones was reported as the main difficulty. That said, the cross‐campus project provided students with exposure to working in an international setting and equipping them with competency for the 21st century highly interconnected workforce (Tan et al., 2017). The only other negative feedback in the study was where students felt that the part of the group located in one campus was taking on more of the load than the other part of the group. A contributing factor to this may have been due to the unexpected effects of coursework deadlines in other modules. For instance, a significant coursework deadline affected the RDHC group meaning their prioritization of the task was much lower than the UNMC group, leaving the UNMC group feeling abandoned with the task. This factor was taken into account in the subsequent years so that both groups had similar accommodations for their workload.

In conclusion, VM has provided an active and interactive way to learn histology and increases enjoyment of a subject students find difficult. Using an e‐workbook approach ensures students participate fully in their own learning. It also fosters collaborative learning, and this keeps students engaged and helps them relate structure to function. Furthermore, the technology has been shown to facilitate group work in an international setting that aided in the development of deep learning of histology as seen by examination performance but also numerous other transferrable skills.

## CONFLICT OF INTEREST

The authors have no conflict of interest to report.

## Data Availability

The data that support the findings of this study are available from the corresponding author upon reasonable request.
